# Demographics and Clinical Characteristics of Patients With Opioid Use Disorder and Offered Medication-Assisted Treatment in the Emergency Department

**DOI:** 10.7759/cureus.41464

**Published:** 2023-07-06

**Authors:** Wayne Fu, Victoria Adzhiashvili, Nima Majlesi

**Affiliations:** 1 Emergency Medicine, Mercy Hospital, Buffalo, USA; 2 Emergency Medicine, Staten Island University Hospital, Staten Island, USA; 3 Psychiatry, Staten Island University Hospital, Staten Island, USA; 4 Medical Toxicology, Staten Island University Hospital, Staten Island, USA

**Keywords:** buprenorphine, substance abuse, drug addiction, medication for opioid use disorder, opioid use disorders

## Abstract

Background and objective

The opioid use disorder (OUD) epidemic is a persistent public health crisis in the United States. Medication-assisted treatment (MAT) with opioid agonists, including buprenorphine, is an effective treatment and is commonly initiated in the emergency department (ED). This study describes the demographics and clinical characteristics of OUD patients presenting to the ED and evaluated for MAT.

Methodology

A retrospective, single-center descriptive study of 129 adult patients presenting to the ED between July 2018 and July 2020 with OUD and evaluated for MAT.

Results

A total of 129 patients were assessed for MAT. About half (53%) received MAT; the remaining received only a referral (35%) or declined any intervention (12%). The median age was 36 years interquartile range (IQR, 28-46 years) and predominantly male (73%), single (65%), white (73%), unemployed (57%) with public insurance (55%), and without a primary care physician (58%). Majority of the patients presented with opioid withdrawal (62%) or intoxication (15%), while 23% presented with other complaints. About half of the patients (51%) were discharged with a naloxone kit. The majority of the patients were induced with buprenorphine with 4 mg or less (54%) and only 6% of patients received repeat dosing.

Conclusions

Male, white patients who are unmarried and unemployed, lack primary care follow-up, and rely on public insurance are more likely to be candidates for MAT. Providers should always maintain a high suspicion of opioid misuse and optimize treatment for those in withdrawal. Understanding these characteristics in conjunction with recent health policy changes will hopefully guide and encourage ED-initiated interventions in combating the opioid crisis.

## Introduction

The opioid crisis continues to be a concerning public health issue in the United States. In 2020, nearly 2.7 million Americans had opioid use disorder (OUD), an increase from 2.0 million individuals in 2018 [1,2]. Consequently, opioid overdose deaths increased from 46,802 deaths in 2018 to 68,630 deaths in 2020.

To combat this major health crisis, the U.S. Food and Drug Administration approved medication-assisted treatment (MAT) including buprenorphine for the treatment of OUD. Buprenorphine is a partial opioid agonist that treats OUD by decreasing withdrawal, craving, and opioid use. Studies have shown that the use of buprenorphine for OUD significantly improves treatment retention, suppression of illicit opioid use, and reductions in all-cause and overdose mortality [3-6]. Despite these benefits and clinical guidelines supporting the use of these medications, 80% of patients do not receive treatment from these life-saving medications [7,8].

To bridge this treatment gap, the emergency department (ED) has become an important clinical setting for screening and identifying patients with OUD. Patients with OUD are at increased risk of adverse health consequences and often seek medical care in the ED, whether it is for their ongoing substance use, or other health issues ranging from psychiatric crises to acute illness or trauma [6]. These interactions in the ED are a critical period for counseling, and treatment through buprenorphine induction and follow-up with outpatient treatment can be an important first step to recovery. Buprenorphine is a safe and effective treatment for OUD that rapidly relieves withdrawal symptoms. When initiated in the ED with referral to outpatient care, MAT promotes cost-effective, addiction treatment engagement [6-9].

While buprenorphine can be prescribed from the ED to continue treatment in the outpatient setting, it is prescribed by less than 5% of primary care physicians [10]. Although overall buprenorphine use has steadily increased over the last decade, there has been a troubling decline in use for individuals aged 15-24 years, which coincided with an increasing rate of opioid-related deaths during this period [11]. Among the many barriers involving buprenorphine treatment was the X-waiver, a requirement imposed by the U.S. Congress under the Drug Addiction Act of 2000 where clinicians were forced to undergo an additional training course to prescribe buprenorphine. Furthermore, there is a lack of consensus and evidence-based strategies for the most effective way of transitioning OUD patients from the ED to appropriate outpatient services [12]. Recent legislation in 2023 eliminated the X-waiver requirement to encourage more widespread use of buprenorphine in the ED and outpatient setting.

This study investigates the demographics and clinical characteristics of OUD patients and those receiving ED-initiated buprenorphine at a single-center, two-campus hospital in Staten Island, New York, one of the epicenters of the opioid crisis. Identification and understanding of these factors may help guide future interventions and initiatives in the ED and provide insight for outpatient community-based programs.

## Materials and methods

This was a retrospective, single-center, chart review study of 129 subjects with OUD who presented to the Staten Island University Hospital ED between November 2018 and July 2020. Staten Island University Hospital is a two-campus 668-bed, tertiary-care teaching hospital. The ED has a combined census of 125,000 patient visits per year between its North (92,000) and South (33,000) campuses. The local Institutional Review Board approved this study.

All subjects were assessed by ED providers and identified as patients with OUD. These patients were then offered a next-day follow-up appointment at an ancillary detoxification clinic, and if clinically indicated, induction with buprenorphine. Repeat evaluations within 24 hours of the first encounter or subjects with incomplete data were excluded from this study. The data of subjects who chose induction and/or follow-up appointment at the ancillary withdrawal clinic were entered by the patients’ ED providers into our hospital Research Electronic Data Capture (REDCap, Vanderbilt University, Nashville, TN, USA) collection software, a secure, web-based application designed to support data capture for research studies.

Two study members trained in the study protocol and data abstraction each reviewed patient records. We utilized a predesigned, standardized case report form to record the data from the electronic chart reports. An additional researcher checked the accuracy of data input for 10% of all subjects to eliminate errors and ensure consistency and accuracy.

Demographic characteristics that were collected included age, gender, insurance status, marital status, and employment status. Clinical characteristics that were collected included primary care physician listed, and emergency room statistics such as mode of arrival, triage level, triage pain rating, and chief complaint. Opioid use characteristics included the type of opioid used and type of intervention received (ED-buprenorphine induction and appointment; appointment only; declined either intervention) were also collected. Finally, intervention-specific characteristics that were collected included the Clinical Opiate Withdrawal Scale (COWS) score of those receiving buprenorphine, total milligrams of buprenorphine given for induction, those receiving repeat dosing, and those receiving naloxone kits at discharge. The COWS is a scoring tool used by clinicians to measure the severity of patients' opioid withdrawal symptoms, categorizing them into mild (5-12 points), moderate (13-24 points), or severe (24+) withdrawal. 

Data collection and processing

The data were analyzed using descriptive statistical methods and were expressed as frequency counts and percentages for categorical variables or as mean and standard deviation or median and interquartile range (IQR), as appropriate, for continuous variables. Data analyses were conducted using Microsoft Excel Version 2019 (Microsoft, Redmond, WA, USA).

## Results

During the study period of November 2018 to July 2020, 132 patient encounters with OUD were identified. Three encounters were excluded, with two patients having incomplete data and one patient undergoing a repeat evaluation within 24 hours. Of the included 129 subjects, 68 (53%) opted for ED-initiated buprenorphine and follow-up appointment, 45 (35%) opted for only a follow-up appointment, and 16 (12%) declined any intervention (Table 1).

**Table 1:** Demographic characteristics of subjects (N = 129). PCP, primary care physician

Demographic characteristics	Appointment only	Buprenorphine induction + appointment	Refused MAT	All groups
Age (median) (years)	36	36	40	36
Q1	28	28	28	28
Q3	46	48	50	48
Gender				
Male	33	48	13	94
Female	12	20	3	35
Marital status				
Single	28	45	11	84
Married	8	8	4	20
Divorced	1	7	0	8
Widowed	0	1	0	1
Unknown	8	7	1	16
Ethnicity				
White	33	47	13	93
Black	6	3	0	9
Hispanic	1	3	1	5
Multiracial	2	5	1	8
Other	1	5	0	6
Unknown	2	5	1	8
Employment status				
Employed	10	22	5	37
Unemployed	31	34	9	74
Disabled	3	3	1	7
Retired	1	9	1	11
Insurance status				
Public	14	48	9	71
Private	30	19	7	56
Self-pay	1	1	0	2
PCP listed				
Yes	18	27	9	54
No	27	41	7	75

The median age was 36 (IQR 28-46), and patients were predominantly male (73%), single (65%), white (73%), unemployed (57%) with public insurance (55%), and without a primary care physician (58%). The predominant age group was 18 to 39 years old (61%). The complete table of demographics for all 129 subjects can be found in Table 1.

Table 2 outlines the characteristics of the patient’s ED courses. Patients were most likely to present with a chief complaint of opioid withdrawal (62%) and drug intoxication/overdose (15%). Less common complaints (23%) included gastrointestinal issues (e.g., abdominal pain, nausea, and vomiting), chest pain, seizure, and other various complaints, including weakness, skin infection, concern for sexually transmitted disease, hyperglycemia, abnormal blood culture, and cough. Patients were most likely to present as walk-ins (50%) and via ambulance (33%). The majority of patients were triaged with a pain rating of 0-3 (70%), and most were seen by providers within the first 30 minutes (83%). Altogether, eight patients (6%) eloped before their ED workup was complete.

**Table 2:** ED course. ED, emergency department

	Appointment only	Buprenorphine induction + appointment	Refused MAT	All groups
Chief complaint				
Gastrointestinal issue	3	4	1	8
Intoxication/Overdose	8	5	6	19
Chest pain	4	4	1	9
Opioid withdrawal	26	50	2	78
Requesting buprenorphine	1	1	0	2
Other	3	4	6	13
Mode of arrival				
Ambulance	14	23	5	42
Private vehicle	8	13	2	23
Walk-in/public transportation	23	32	9	64
Triage level				
Triage 1	0	0	0	0
Triage 2	7	9	4	20
Triage 3	33	52	12	97
Triage 4	5	7	0	12
Triage pain rating				
0-3	32	47	11	90
4-7	7	15	4	26
8-10	4	6	1	11
Unknown	2	0	0	2

Table 3 describes the specific opioid etiology of patients and their management in the ED. Heroin was the most commonly identified opioid (52%) followed by oxycodone (18%). The majority of patients used opioids within the last day (54%). Of the 68 patients receiving MAT, patients were most likely to be in mild (40%) or moderate (38%) withdrawal and induced with 4 mg or less (54%) or between 4 and 8 mg of buprenorphine (35%), with only four patients (6%) receiving repeat dosing. Half of the patients receiving MAT (50%) were documented to have a reassessment COWS score. About half of the patients were discharged with an at-home naloxone kit (51%).

**Table 3:** Opioid use and treatment characteristics. COWS, Clinical Opiate Withdrawal Scale; MAT, medication-assisted treatment

	Appointment only	Buprenorphine induction + appointment	Refused MAT	All groups
Type of opioid used				
Dilaudid	0	1	0	1
Fentanyl	2	1	0	3
Heroin	24	32	8	64
Heroin and oxycodone	1	1	0	2
Kratom	1	1	0	2
Methadone	0	3	1	4
Morphine	1	3	0	4
Oxycodone	7	13	1	21
Suboxone	1	9	0	10
Tramadol	0	1	0	1
Unknown	8	3	6	17
Last used opioid (days)				
0-1	28	32	9	69
2-4	4	22	1	27
5-7	4	2	0	6
7+	1	3	0	4
Unknown	10	6	7	23
COWS score of those receiving MAT				
No withdrawal (0-4)		7		
Mild withdrawal (5-12)		27		
Moderate withdrawal (13-14)		26		
Severe withdrawal (15+)		4		
Unknown		4		
Amount of buprenorphine induction (g)				
≤4		37		
4-8		24		
8+		7		
Pt was given a Narcan kit				
Yes	17	46	3	66
No	28	50	13	63

## Discussion

OUD remains a major public health problem in the United States. ED-initiated buprenorphine and subsequent referral to outpatient care are safe and cost-effective treatments. This study summarizes the demographics and clinical characteristics of patients with OUD in the ED.

Over half the patients with OUD opted for buprenorphine treatment, while the remaining accepted a follow-up appointment or declined intervention altogether. Weicker et al. investigated the willingness of patients to try buprenorphine and identified common reasons for apprehension to buprenorphine, including satisfaction with current agonist therapy (e.g., methadone) and unfamiliarity with buprenorphine [13]. Only about half of the patients in our study were discharged with a naloxone kit suggesting that patients may not be fully aware of the potential dangers of opioid use and the value of this potentially life-saving antidote. Targeted educational interventions to increase awareness and understanding of buprenorphine and opioid use may increase the willingness of patients to take buprenorphine or receive outpatient follow-up.

The subjects in this study were predominantly white, single males, adults aged 18-39 years on public insurance with no employment. This trend follows prior studies that show that young, white males are the most commonly identified opioid users [14-16]. In addition, marriage and employment are a protective factors in patients with OUD, including higher long-term recovery rates, lower risk of early treatment discontinuation, and better quality of life, placing unmarried patients further at risk [17-19]. While whites appear to be the most vulnerable to opioid abuse, recent studies suggest that mortality rates from opioid use in non-white minorities have also been significantly rising over the last 15 years suggesting that the opioid misuse treatments should target all patients regardless of race [20].

Over half of the patients did not have a primary care physician putting them at risk for lack of follow-up care. D'Onofrio et al. demonstrated the impact of primary care follow-up in significantly improving addiction treatment engagement and reduction of illicit opioid use [6]. This study also identified that nearly 60% of the chief complaints were for opioid withdrawal and 15% represented drug intoxication/overdose. Triage notes that directly mention opioid involvement can potentially alert ED providers in identifying patients that may benefit from opioid abuse treatment. However, the remaining 23% of patients had chief complaints such as chest pain or abdominal pain that were not as initially obvious for patients at risk for opioid misuse. Moreover, triage notes for intoxication/overdoses may not directly specify opioid use, especially when these patients are not conscious enough to provide clear information. The majority of patients were triaged with a low pain rating (0-3) despite the known debilitating effects of opioid withdrawal and heightened pain sensitivity in opioid users [21,22]. Given the known pitfalls of triage bias and anchoring in the ED and the wide prevalence of OUD in the United States, providers should always have a high suspicion of opioid abuse regardless of the chief complaint or triage note [23].

Heroin was the most commonly identified opioid used followed by oxycodone. While prescription opioids (e.g., oxycodone) remain the most commonly abused opioid in the US, opioid-related deaths continue to rise and are caused primarily by heroin and synthetic opioids such as fentanyl [24]. Patients were most likely to come in within the first day after opioid use, and almost three-quarters of patients presented within the first four days. This is not surprising given the onset of withdrawal symptoms of the majority of opioids are within 6-12 hours and peak around the first 24-72 hours prompting individuals to seek medical care [24].

Patients receiving MAT were most likely to be identified in mild or moderate withdrawal (COWS 8-24). Interestingly, 10% of patients were identified with minimal withdrawal (COWS 0-4) but still started on buprenorphine despite institution guidelines only recommending induction of patients with COWS scores above 8. This may have been performed either due to lack of understanding of treatment guidelines, or anticipation of worsening symptoms. Furthermore, only four out of the 68 patients received a repeat dose, and the majority received only 4 milligrams of buprenorphine or less. There is some evidence that most patients with OUD require higher doses for effective suppression of withdrawal symptoms, and doses even greater than 12 milligrams can be safe and effective [25-27]. Patients may have also benefitted from reassessment after treatment with repeated COWS scores to indicate improvement of their withdrawal symptoms or if they required another dose of buprenorphine. However, given that only half the patients had a documented reassessment COWS score, it is unclear if patients were sufficiently treated for their withdrawal or if there was simply inadequate reassessment of the patient after their first dose of buprenorphine. Lastly, only about half of the patients were discharged with an at-home naloxone kit. Studies have shown that the use of take-home naloxone kits can reduce overdose mortality and repeat ED visits, but its distribution remains low [28].

Recent legislation eliminating the X-waiver will hopefully reduce barriers to access to buprenorphine and ideally promote the use of this life-saving treatment in both ED and outpatient setting. Furthermore, clarification of buprenorphine treatment guidelines, careful reassessment and monitoring of the patient’s symptoms, and adequate dosing of buprenorphine may increase the effectiveness of MAT in OUD patients.

Limitations

Our study has several limitations. This was a descriptive study conducted at a single center without a comparison group, and its findings cannot be generalized to all OUD patients or show any causality. Due to the retrospective nature of our study, specific patient data was sometimes incomplete or missing. Moreover, our data relied primarily on information reported by providers and patients during their ED visits and can vary between clinicians. For example, elements of the COWS score can be subjective; scoring for certain withdrawal symptoms such as lacrimation and sweating can vary based on how severe the clinician perceives these symptoms. Other confounding variables were not captured in this study including socioeconomic status, preexisting medical conditions, and other substance use. Data were also at risk for sampling bias because providers were required to identify patients based on their clinical assessment. Other characteristics such as the type of opioid used or the last time since the last opioid was used rely on the patient’s self-reporting and are subject to recall and reporting bias.

## Conclusions

OUD continues to be a prevalent problem in the United States. Understanding the demographics and clinical characteristics of OUD patients and those receiving ED-initiated buprenorphine can help guide future intervention efforts in the ED. In this patient population, we observed they were more likely to be young adult, white unemployed males without primary care follow-up. Heroin continues to be the most commonly misused opioid seen in patients presenting to the ED and patients in opioid withdrawal may benefit from careful assessment of symptoms and optimal dosing of buprenorphine. ED providers should maintain a high suspicion of opioid misuse in all patients regardless of the chief complaint or triage note. Better educational awareness from both providers and patients, in addition to ongoing health policy reforms, will hopefully improve access to MAT and reduce barriers to helping communities recover from the devastating opioid use epidemic.
